# Non-invasive Amide Proton Transfer Imaging and ZOOM Diffusion-Weighted Imaging in Differentiating Benign and Malignant Thyroid Micronodules

**DOI:** 10.3389/fendo.2018.00747

**Published:** 2018-12-12

**Authors:** Ruijian Liu, Guihuang Jiang, Peng Gao, Guoming Li, Linghui Nie, Jianhao Yan, Min Jiang, Renpeng Duan, Yue Zhao, Jinxian Luo, Yi Yin, Cheng Li

**Affiliations:** ^1^Department of General Surgery, Guangdong Second Provincial General Hospital, Guangzhou, China; ^2^Department of Medical Imaging, Guangdong Second Provincial General Hospital, Guangzhou, China; ^3^Guangdong Traditional Medical and Sports Injury Rehabilitation Research Institute, Guangdong Second Provincial General Hospital, Guangzhou, China

**Keywords:** thyroid nodule, diagnosis, magnetic resonance imaging (MRI), amide proton transfer (APT), diffusion-weighted imaging (DWI)

## Abstract

**Background:** Pre-operative non-invasive differentiation of benign and malignant thyroid nodules is difficult for doctors. This study aims to determine whether amide proton transfer (APT) imaging and zonally oblique multi-slice (ZOOM) diffusion-weighted imaging (DWI) can provide increased accuracy in differentiating benign and malignant thyroid nodules.

**Methods:** This retrospective study was approved by the institutional review board and included 60 thyroid nodules in 50 patients. All of the nodules were classified as malignant (*n* = 21) or benign (*n* = 39) based on pathology. It was meaningful to analyze the APT and apparent diffusion coefficient (ADC) values of the two groups by independent *t*-test to identify the benign and malignant thyroid nodules. The relationship between APT and ZOOM DWI was explored through Pearson correlation analysis. The diagnostic efficacy of APT and ZOOM DWI in determining if thyroid nodules were benign or malignant was compared using receiver operating characteristic (ROC) curve analysis.

**Results:** The mean APTw value of the benign nodules was 2.99 ± 0.79, while that of the malignant nodules was 2.14 ± 0.73. Additionally, there was a significant difference in the APTw values of the two groups (*P* < 0.05). The mean ADC value of the benign nodules was 1.84 ± 0.41, and was significantly different from that of the malignant nodules, which was 1.21 ± 0.19 (*P* < 0.05). Scatter point and Pearson test showed a moderate positive correlation between the APT and ADC values (*P* < 0.05). The ROC curve showed that the area under the curve (AUC) value of ZOOM DWI (AUC = 0.937) was greater than that of APT (AUC = 0.783) (*P* = 0.028).

**Conclusion:** APT and ZOOM DWI imaging improved the accuracy of distinguishing between benign and malignant thyroid nodules. ZOOM DWI is superior to APTw imaging (*Z* = 2.198, *P* < 0.05).

## Introduction

Thyroid nodules are comm and are found in up to 65% of the general population (1). Surgeons and radiologists are often asked to distinguish between benign and malignant thyroid nodules because it is necessary to be aware of the difference when creating treatment plans and surgical strategies, or when performing conservative monitoring of treatment. Examinations of suspected nodules in the clinic are performed using ultrasound, computed tomography (CT), magnetic resonance imaging (MRI), and positron emission tomography (PET). New iterations of the aforementioned imaging techniques have also been integrated into clinical practice including ultrasound contrast, and perfusion or enhanced CT, The use of ^18^F-FDG in diffusion-weighted imaging (DWI) and PET/CT has been proposed as a useful tool for the distinguishing benign from malignant thyroid nodules (2–5). However, all these techniques are hampered by a number of limitations. For example, ultrasound has insufficient power for retrosternal goiters. Fine needle aspiration biopsy (FNAB) is highly sensitive and specific, and it is commonly used to identify benign and malignant thyroid nodules (5). However, it is an invasive test that can cause physical discomfort or pain for patients (1), with a 10–15% rate of non-definitive diagnosis (6). CT uses radiation and is not suitable for pregnant women or adolescents. 18FDG-PET uptake in thyroid nodules confirmed by ultrasonography increases the risk of thyroid cancer (5). Contrast-enhanced MRI is forbidden for patients with renal failure and contrast media allergy (7). Therefore, a non-invasive and economical method is urgently needed in the clinic to detect and discern between benign and malignant thyroid nodules.

Amide proton transfer (APT) imaging is a molecular MRI method based on chemical exchange saturation transfer that can be used to detect endogenous mobile proteins and peptides even at relatively low molecular concentrations (8–13). Protein accounts for approximately 18% of the human body weight and performs most cellular functions. These proteins can be divided into two types: semi-solid proteins and mobile proteins. Mobile proteins are the basis of APT imaging. APT imaging has been introduced in the clinic for the imaging of breast cancer (14, 15), brain tumors (9, 10, 13), rectal cancer (16), lung cancer (17, 18), prostate cancer (19, 20), and non-neoplastic diseases, such as stroke (21, 22) and ventral hernia (23). Previous studies have shown that APT imaging, as an MRI biomarker for malignant tumors, can help identify the most active proliferative components in the tumor and predict the response of the tumor to treatment. Although APT imaging has had a positive effect on the diagnosis of diseases, to date, APT images have not been developed for or applied to thyroid nodules.

DWI obtains image contrast by measuring the degree of freedom and diffusion direction of water molecules in tissue (24). The apparent diffusion coefficient (ADC) is an important parameter of DWI images (25), which are commonly used in the diagnosis of the thyroid gland (26). Zonally oblique multi-slice (ZOOM) imaging is a novel DWI imaging method. The ZOOM acquisition method provides better image quality and accuracy than non-ZOOM technology (27). It has been proven that it can be applied to the diagnosis of other diseases by scanning areas of the body such as the cervical spinal cord. A shorter time of repetition (TR) can be obtained when ZOOM DWI is used, along with better image quality, higher blood contrast, and less magnetically sensitive artifacts.

In this study, 60 thyroid nodules were obtained from 50 patients who underwent preoperative MRI. The hypothesis of this confirmatory study is that the two non-invasive advanced MRI techniques, free protein-based APT imaging and water-based molecular diffusion-based ZOOM DWI, can be valuable in differentiating between benign and malignant thyroid nodules. Additionally, ZOOM DWI is superior to APTw imaging.

## Materials and Methods

### Study Population

**Figure 1:**
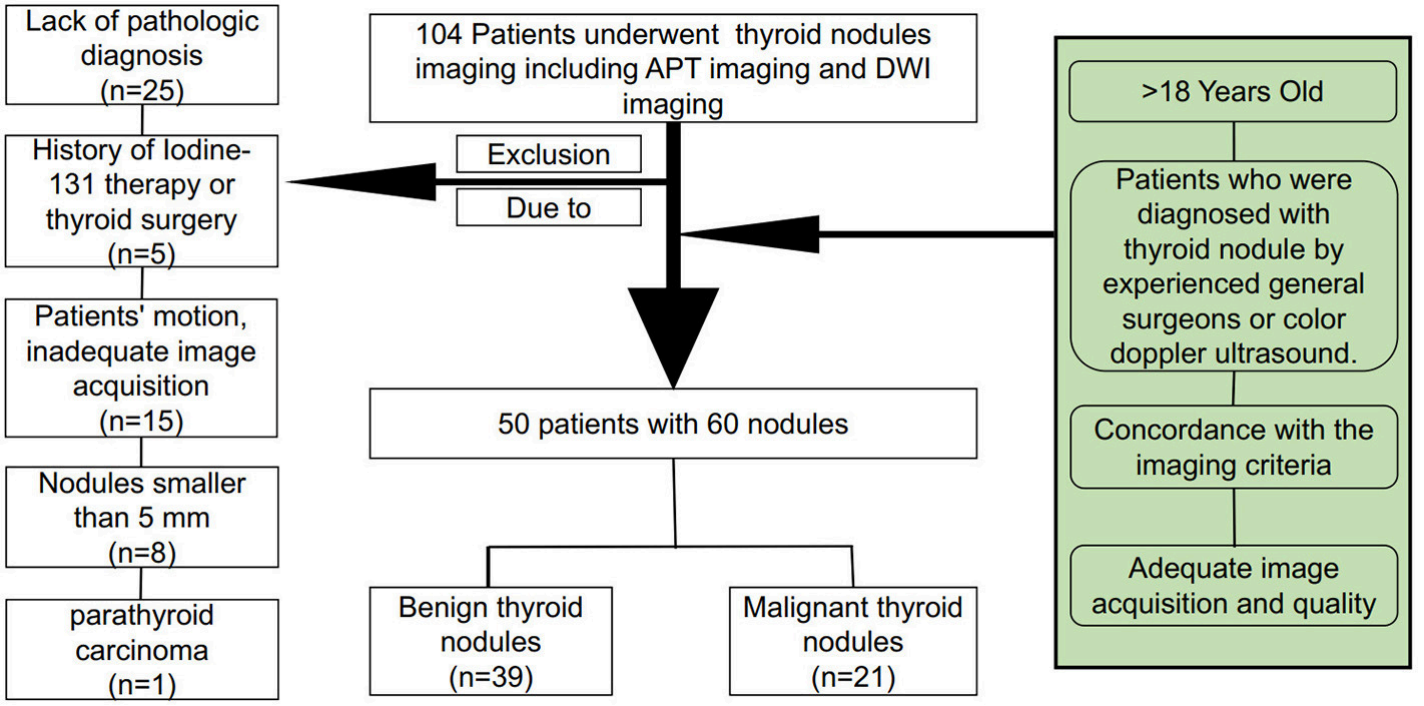
Flowchart of the patient inclusion and exclusion process.

This study was approved by the Ethics Committee of Guangdong Second People's Hospital. All of the patients signed written informed consent forms prior to inclusion. All of the patients underwent conventional short tau inversion recovery (STIR), T1-weighted and T2-weighted imaging. The latest ZOOM DWI and APTw imaging sequences from *in vitro* MR images for pre-treatment thyroid nodule evaluation from November 2017 to May 2018 were used in the current study. Among 104 patients, 25 patients were excluded because they had no pathological diagnosis due to refusing surgery or undergoing conservative treatment. Five cases of thyroid ^131^I treatment history or thyroid surgery history were excluded. In the remaining 74 patients, 15 who showed substantial patient motion and inadequate image acquisition were excluded, 8 were excluded because of nodules smaller than 5 mm, and 1 case was excluded because of pathological diagnosis of parathyroid carcinoma. Finally, a total of 50 patients with 60 nodules, including 39 benign thyroid nodules and 21 malignant thyroid nodules, were enrolled in this study, as shown in Figure 1.

All of the cases were diagnosed by pathology after surgery. The histologic types of thyroid nodules were as follows: 20 thyroid papillary carcinomas, 1 follicular thyroid cancer, 17 nodular goiter, 20 thyroid adenomas, and 2 cases of Hashimoto's thyroiditis. All of the patients underwent magnetic resonance imaging within 3 days before surgery.

### MRI Protocols

All of the cases were subjected to magnetic resonance imaging of the thyroid on a Philips 3T whole-body scanner (Ingenia, 3.0 T; Philips Medical Systems, The Netherlands) with a bore size of 70 cm. In this study, magnetic susceptibility artifacts were reduced in all cases by having patients remove any metal dentures before magnetic resonance imaging. Motion artifacts were reduced by training patients to hold their breath at the appropriate times and avoiding swallowing. A sensitivity-encoding 16-multichannel receiver for head and neck coils was used for thyroid scanning. Our scanning protocol used these sequences to obtain axial imaging: STIR, T1W, T2W, ZOOM DWI, APT, and Gd-enhanced T1-weighted (Gd-T1W) imaging. Gadopentetate dimeglumine (0.2 ml/kg body weight; Magnevist; Bayer Schering, Guangzhou, China) was injected as a bolus via an antecubital vein at a speed of 2.0 mL/s to acquire the axial Gd-T1W images. Specific parameters are given in Table 1.

**Table 1:** Details of the MR parameters.

**PARAMETER**	**APT**	**STIR**	**ZOOM DWI**	**T1W**	**T2W**
FOV(mm)	212 × 182 × 44	220 × 220 × 92	116 × 51 × 79	230 × 218 × 108	220 × 230 × 108
voxel	1.8 × 1.8 × 4.4	0.9 × 0.9 × 4	1.81 × 1.81 × 4	0.9 × 1.1 × 4	0.9 × 1.1 × 4
Matrix	120 × 101 × 10	244 × 219 × 21	64 × 27 × 18	256 × 192 × 24	224 × 200 × 24
NSA	1	1	1	2	2
Fat saturation	SPIR	NO	SPAIR	NO	No
TE	5.9	90	62	18	100
TR	6820	2367	3687	570	2500
Reconstruction matrix	224	400	128	480	528
TSE	158	25	NO	60	23
Flig angle	90	NO	90	90	90
Total time(s)	259	161	221	85	150

The scan time for each sequence was limited to 5 min, and the imaging acquisition time was intentionally minimized to alleviate patient discomfort. Therefore, gating and anesthesia were not used during the scan. The patient was trained to avoid movement and swallowing before scanning to reduce motion artifacts and improve the image quality.

### Image Analysis

The radiologist (Guihua Jiang) and thyroid surgeon (Cheng Li) analyzed each patient's APT and ZOOM DWI images based on post-operative pathology and intra-operative findings. To compare the APT imaging with ZOOM DWI, we analyzed all of the image data using prototype software developed by Philips Medical Systems. The definitions and terms used in this study have been described in previous studies (11, 12, 28–33). APT imaging was quantified by magnetization transfer ratio asymmetry (MTRasym) analysis. The calculated MTRasym (3.5 ppm) image, using B0-corrected magnetization transfer spectral data at the offset of ±3.5 ppm, was deemed the APT image. Philips post-processing software was used to automatically generate ADC diagrams from ZOOM DWI images. The ADC value was automatically calculated after the region of interest (ROI) of each nodule on the ADC map was delimited.

For each imaging method, a combination of surgical findings and pathological results were used. Each ROI was drawn by 1 thyroid surgeon (Cheng Li) and 1 radiologist (Guihua Jiang), who together have 20 years of experience. For each patient, based on the STIR, T1W, and T2W imaging, and enhanced imaging, the ROIs were drawn on the APT imaging and ZOOM DWI images (after removing areas of necrosis, hemorrhage, calcium deposition, and cysts). Each ROI was maximized by placing it at the focus of the entire cross-sectional area of the nodule, thereby minimizing the effects of non-uniformity of the nodule. To minimize the error, each lesion was measured 3 times, and the values averaged to determine the ADC and APT.

### Statistical Analysis

In this study, SPSS 20.0 and MedCalc 15 software were employed for data analysis. The relationship between the molecular parameters, the APTw, and ADC values between different benign and malignant thyroid nodules were analyzed by two-samples *t*-tests. APTw and ADC were statistically evaluated by Pearson correlation. A comparison of diagnostic effects of APT and ZOOM DWI imaging was performed using the receiver operating characteristic (ROC) curve analysis of MedCalc 15. *P* < 0.05 was considered statistically significant.

## Results

### Demographics and Clinical Characteristics

No significant differences were observed in age, gender distribution, height, or weight between individuals with benign thyroid nodules and those with malignant nodules. Patient demographic data and characteristics are shown in Table 2.

**Table 2:** Histological categories and demographic data between benign thyroid individuals and malignant thyroid individuals.

**Histological categories**	**Number (%)**	**Mean age(years)**	**Female (%)**	**Height (m)**	**Weight (kg)**
Benign	39 (65)	43.46 ± 12.79	27 (69.2)	1.62 ± 0.07	60.96 ± 9.13
Thyroid adenoma	20 (33.3)	38.55 ± 11.42	13 (65)	1.64 ± 0.08	59.05 ± 8.86
Nodular goiter	17 (28.3)	50.76 ± 10.9	13 (76.5)	1.59 ± 0.07	62.5 ± 8.06
Hashimoto's thyroiditis	2 (3.3)	30.5 ± 10.61	1 (50)	1.63 ± 0.07	67 ± 21.21
Malignant	21 (35)	39.86 ± 13.08	15 (71.4)	1.61 ± 0.07	59.1 ± 10.71
Thyroid papillary carcinoma	20 (33.3)	38.65 ± 12.16	14 (70)	1.61 ± 0.07	58.9 ± 10.95
Follicular thyroid cancer	1 (1.7)	64	1 (100)	1.56	63
Total	60 (100)	42.2 ± 12.9	42 (70)	1.61 ± 0.07	60.31 ± 9.66

### Data Analysis of APT Imaging and Zoom DWI

**Figure 2:**
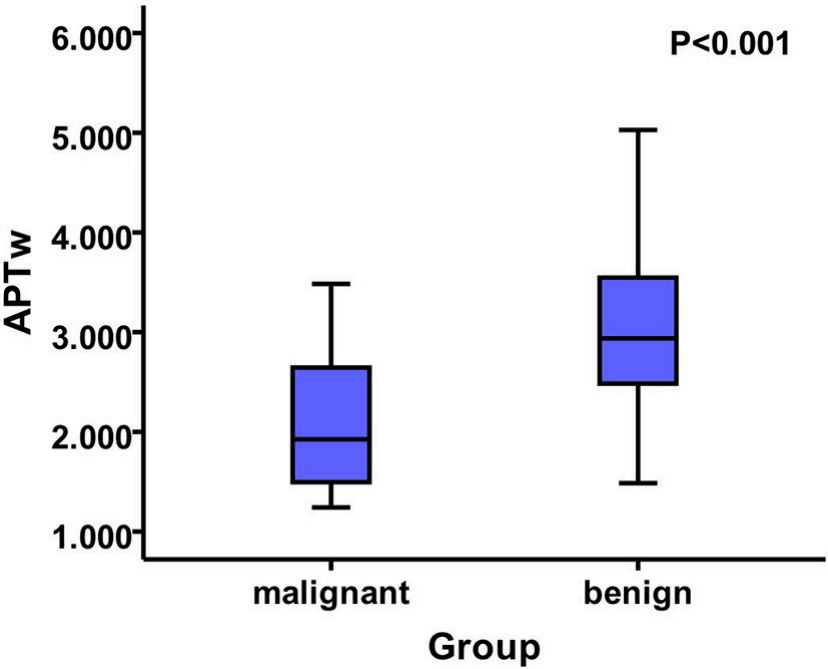
The mean APTw value of the benign nodules was 2.99 ± 0.79, while that of the malignant nodules was 2.14 ± 0.73. Additionally, there was a significant difference between the two groups of APTw values (*P* < 0.05).

**Figure 3:**
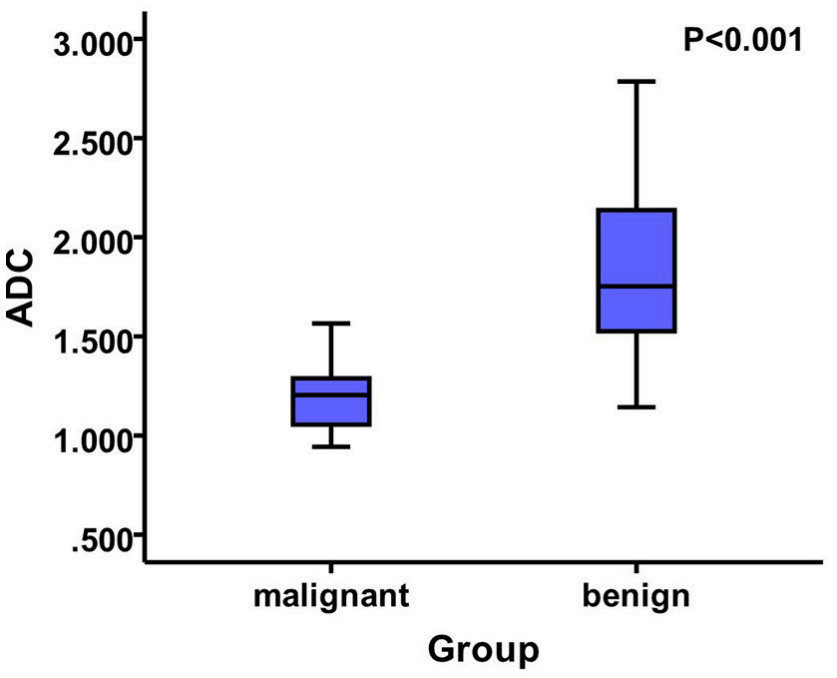
The mean ADC value of the benign nodules was 1.84 ± 0.41, whereas that of the malignant nodules was 1.21 ± 0.19 that showed significant difference among them (*P* < 0.05).

The two-sample *t*-test analysis revealed significantly increased APT values for benign thyroid nodules in comparison to APT values for malignant thyroid nodules (*P* < 0.05) (Figure 2). Additionally, the ADC of malignant thyroid nodules was significantly lower than that of benign nodules (*P* < 0.05) (Figure 3).

### Correlation Between APTw and ADC

**Figure 4:**
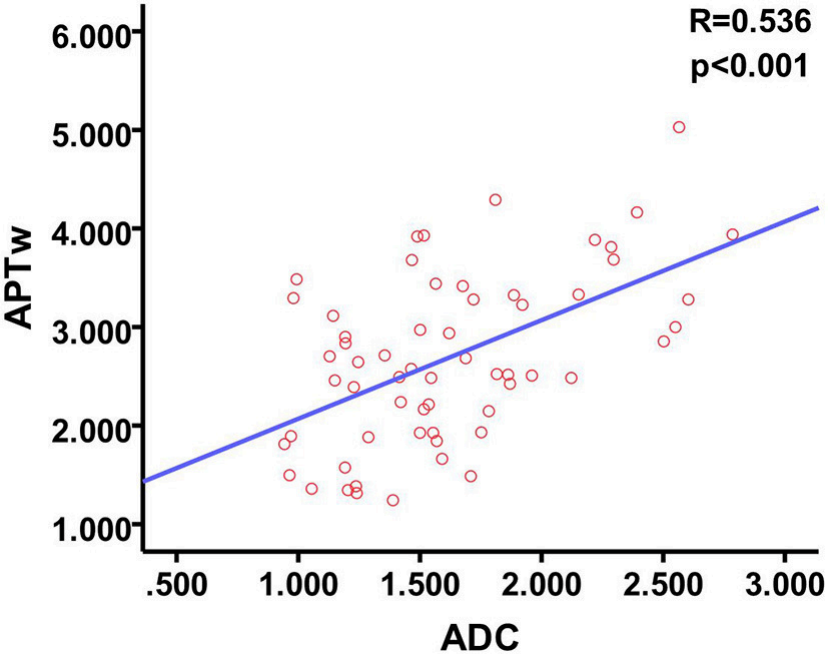
Scatter plot and Pearson test show a moderate positive correlation between APTw and ADC.

There was a positive correlation between the mean ADC observed in patients with malignant thyroid nodules and the APTw (*P* < 0.001, *R* = 0.536) (Figure 4).

### Comparison of the Diagnostic Performance Between APT Imaging and ZOOM DWI Imaging

**Figure 5:**
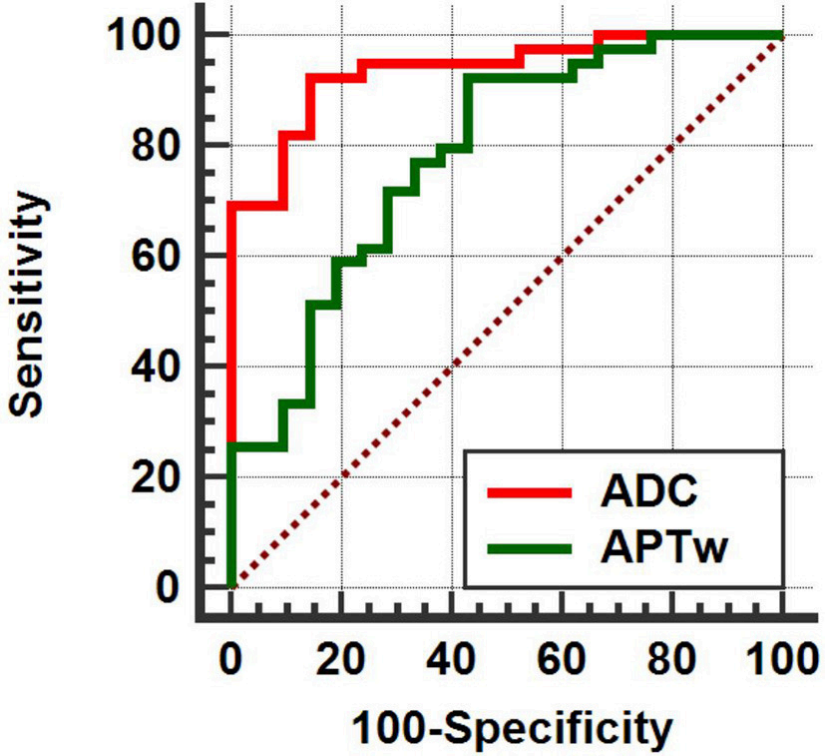
APT (AUC = 0.783) < ADC (AUC = 0.937), (*Z* = 2.198, *P* = 0.028).

**Based on** the ROC curve of the APT value (area under the curve, AUC = 0.783) and the ADC value (AUC = 0.937), ZOOM DWI imaging was superior to APT imaging in differentiating **between** benign and malignant thyroid nodules (Figure 5).

## Discussion

Thyroid doctors cannot formulate the most optimal treatment strategies unless accurate evaluation of benign and malignant thyroid nodules can be performed using non-invasive magnetic resonance imaging. APT imaging is a molecular non-invasive MRI technique based on the chemical exchange saturation transfer (CEST) mechanism that detects endogenous mobile proteins and peptides in biological tissues. In this study, we put forward for the first time that APT imaging is meaningful for the differential diagnosis of benign and malignant thyroid nodules. The APT signal of malignant thyroid nodules is lower than that of benign nodules. Malignant thyroid nodules had lower ADC signal values than those of benign thyroid nodules. In addition, we found a moderate positive correlation between APT values and ADC values. Lastly, there were no significant differences between ZOOM DWI imaging and APT imaging in the diagnosis of benign and malignant thyroid nodules.

In our study, all of our diagnoses came from pathological assessment after thyroidectomy, which is a more accurate reference. In this study, we chose >5 mm nodules rather than >10 mm because we used the latest imaging sequences and an advanced imaging system (Philips 3.0T MRI), in order to obtain the best quality images. Additionally, we reduced imaging artifacts by training patients to remain as immobile as possible during scans.

To date, there have been no studies that have examined the sensitivity and specificity of APT imaging for thyroid nodules. Our APT imaging study of the thyroid is different from similar studies that have examined other parts of the body. Previous studies have shown that APT signals are higher in malignant nodules or malignant tumors of other organs. For example, the APTw values of malignant brain tumors were higher than those of benign brain tumors (9, 11, 12), the APTw values of high-grade gliomas were higher than those of low-grade gliomas (10, 13, 30, 34), the APTw values of moderately differentiated colorectal cancer were higher than those of highly differentiated colorectal cancer (16), and the APTw values for malignant pulmonary lesions were higher than those of benign pulmonary lesions (35). However, there have also been differences in studies, and the APTw value of the prostate cancer Gleason score (GS) in the score 7 group was the highest among the score 6–9 groups (19).

Unlike other malignancies, papillary thyroid cancer is a very latent disease (36–39), Therefore, the slow proliferation of cells accounts for the lower APTw value. In contrast, thyroid adenomas often grow into solitary large nodules, and the nodular goiter is diffusely enlarged. This phenomenon is in line with the principle of APT imaging, which is mainly based on the concentration of free proteins and peptides in the thyroid nodules. The APTw value increases with increases in the concentration of mobile proteins and peptides (40–42). Malignant nodules may destroy the thyroid membrane, thereby affecting the transport and exchange of related microscopic substances. Studies have confirmed by pathology and contrast-enhanced ultrasound that the microvessel density of malignant thyroid nodules is lower than that of benign thyroid nodules. The low microvessel density limits proton exchange between free protein and water, reduces cell growth and metabolism, and results in malignant thyroid nodules. In our study, APT values were lower in malignant thyroid nodules than in benign thyroid nodules. In addition, the nuclei of malignant thyroid nodules are larger, and the proportion of nuclear cytoplasm is increased (43). This may be one reason to account for the decreased APT values in malignant thyroid nodules in comparison to benign thyroid nodules.

Our findings on ZOOM DWI in thyroid nodules are consistent with those of previous studies, where ADC signals for malignant thyroid nodules are lower than those of benign nodules (25, 26, 44, 45), indicating that the diffusion of water molecules in malignant thyroid nodules is limited. The reduction in microvessel density and the destruction of the thyroid capsule, which occur in thyroid cancer, can also account for lowered ADC signals in malignant thyroid nodules. However, a small number of studies have demonstrated that the differentiation of benign and malignant thyroid nodules based on ADC is ineffective (46). These differences between studies can be ascribed to the different technologies used, including the choice of B values, and the workstations used in ADC computing. The presence of necrotic or hemorrhagic nodules or poor image quality caused by low-field MRI magnets may also account for this difference (39). In addition, we found that there is a moderate positive correlation between APTw values and ADC values. It is possible that both values are related to the exchange of substances, and subsequently increase with increases in the rate of exchange of substances. It was found that ZOOM DWI was superior to APT in the diagnosis of thyroid nodules, which may be explained by APT imaging is a new technology that is not yet fully developed.

This study has several limitations. First, the sample size of the study is relatively small. Future studies will require a larger sample size so as to improve the power of the statistical analysis of our results. In addition, local absorption of heat causes elevated body temperature and results in patients feeling uncomfortable during the examination. Therefore, the specific absorption rate should be considered, which requires the selection of suitable pulse energy and scanning time so that the APT sequence can be further optimized.

## Conclusions

In the current study, APT and ZOOM DWI were compared as two promising imaging methods for evaluating thyroid nodules at the cellular and molecular level. Our results indicate that they provide additional accurate clinical diagnostic information. These important auxiliary methods are of great value for differentiating benign and malignant thyroid nodules. We also found that APT imaging was moderately correlated with ADC values for thyroid nodules.

## Author Contributions

CL designed experiments; GL, LN, and JY carried out experiments; MJ analyzed experimental results; RD analyzed sequencing data and developed analysis tools. YZ, JL, and YY assisted with collecting data; RL, GJ, and PG wrote the manuscript.

### Conflict of Interest Statement

The authors declare that the research was conducted in the absence of any commercial or financial relationships that could be construed as a potential conflict of interest.
